# A nanobody targeting the F-actin capping protein CapG restrains breast cancer metastasis

**DOI:** 10.1186/bcr3585

**Published:** 2013-12-13

**Authors:** Katrien Van Impe, Jonas Bethuyne, Steven Cool, Francis Impens, David Ruano-Gallego, Olivier De Wever, Berlinda Vanloo, Marleen Van Troys, Kathleen Lambein, Ciska Boucherie, Evelien Martens, Olivier Zwaenepoel, Gholamreza Hassanzadeh-Ghassabeh, Joël Vandekerckhove, Kris Gevaert, Luis Ángel Fernández, Niek N Sanders, Jan Gettemans

**Affiliations:** 1 Department of Biochemistry, Faculty of Medicine and Health Sciences, Ghent University, A. Baertsoenkaai 3, B-9000 Ghent, Belgium; 2 Laboratory of Gene Therapy, Faculty of Veterinary Medicine, Ghent University, Heidestraat 19, B-9820 Merelbeke, Belgium; 3 Department of Medical Protein Research, VIB, B-9000 Ghent, Belgium; 4 Department of Microbial Biotechnology, Centro Nacional de Biotecnología, Consejo Superior de Investigaciones Científicas (CSIC), Campus Cantoblanco, 28049 Madrid, Spain; 5 Department of Radiation Oncology and Experimental Cancer Research, Ghent University, Faculty of Medicine and Health Sciences, B-9000 Ghent, Belgium; 6 Pathology Department, Ghent University Hospital, De Pintelaan 185, B-9000 Ghent, Belgium; 7 Nanobody Service Facility, VIB, Brussels, Belgium

## Abstract

**Introduction:**

Aberrant turnover of the actin cytoskeleton is intimately associated with cancer cell migration and invasion. Frequently however, evidence is circumstantial, and a reliable assessment of the therapeutic significance of a gene product is offset by lack of inhibitors that target biologic properties of a protein, as most conventional drugs do, instead of the corresponding gene. Proteomic studies have demonstrated overexpression of CapG, a constituent of the actin cytoskeleton, in breast cancer. Indirect evidence suggests that CapG is involved in tumor cell dissemination and metastasis. In this study, we used llama-derived CapG single-domain antibodies or nanobodies in a breast cancer metastasis model to address whether inhibition of CapG activity holds therapeutic merit.

**Methods:**

We raised single-domain antibodies (nanobodies) against human CapG and used these as intrabodies (immunomodulation) after lentiviral transduction of breast cancer cells. Functional characterization of nanobodies was performed to identify which biochemical properties of CapG are perturbed. Orthotopic and tail vein *in vivo* models of metastasis in nude mice were used to assess cancer cell spreading.

**Results:**

With G-actin and F-actin binding assays, we identified a CapG nanobody that binds with nanomolar affinity to the first CapG domain. Consequently, CapG interaction with actin monomers or actin filaments is blocked. Intracellular delocalization experiments demonstrated that the nanobody interacts with CapG in the cytoplasmic environment. Expression of the nanobody in breast cancer cells restrained cell migration and Matrigel invasion. Notably, the nanobody prevented formation of lung metastatic lesions in orthotopic xenograft and tail-vein models of metastasis in immunodeficient mice. We showed that CapG nanobodies can be delivered into cancer cells by using bacteria harboring a type III protein secretion system (T3SS).

**Conclusions:**

CapG inhibition strongly reduces breast cancer metastasis. A nanobody-based approach offers a fast track for gauging the therapeutic merit of drug targets. Mapping of the nanobody-CapG interface may provide a platform for rational design of pharmacologic compounds.

## Introduction

Aberrant turnover of the actin cytoskeleton is intimately associated with cancer cell migration and invasion. A large number of actin-associated proteins act as downstream executioners of signals integrated by a.o. small GTPases of the Rho family [1]. Causal relations have been established between perturbed expression, subcellular localization or activity of many actin-associated proteins, and cancer cell invasion. Hence, as in many other research areas, actin-regulating proteins are being proposed as new potential targets for drug development at a swift pace. Such targets include factors that promote actin polymerization, such as Arp2/3 and formin [2] or the actin-bundling proteins fascin, filamin-A, and Mena [3], to mention only a few. Alternatively, proteins residing in structures like invadopodia (N-WASp, cortactin) [4], or filopodia (Ena/VASP proteins) [5] are considered to be possible targets of interest. These structures contribute to cell-membrane protrusion and/or enhanced focal metalloprotease activity, leading to local degradation of the extracellular matrix, with ensuing invasion of the surrounding tissue. Thus, cytoskeletal components may constitute a plentiful source of potential targets for further therapeutic development.

However, two important issues slow the progress in this field: the apparent redundancy of the actin system and the lack of tools to study this in a specific manner at the protein level. The sheer number of actin-associated proteins (>100) has led researchers to propose that some functions of actin-associated proteins are redundant, and this is supported by experimental studies. For instance, a double knockout of CapG and gelsolin (two proteins with actin filament-capping activity) shows only mild defects [6], suggesting that the capping function is redundant during development [7]. Other proteins like twinfilin, Eps8, and CapZ, also display capping activity.

Notwithstanding these findings, it should be emphasized that an overwhelming lack of specific inhibitors (targeting cytoskeletal constituents) allows scrutiny of genetic data at the protein level.

CapG binds reversibly to the barbed end of actin filaments (F-actin capping) in a calcium-dependent manner [8]. Elevated CapG levels enhance cellular motility/chemotaxis [9] and are associated with increased invasion into collagen type I or chick heart fragments [10]. Conversely, downregulation of CapG expression reduces invasion of various cancer cell lines [11-13]. In recent years, several proteomic studies demonstrated that CapG is overexpressed in various types of cancer [14-16], including breast cancer. Interestingly, higher expression of CapG was observed in the tumor margin where invasive cells are located, pointing to a role for CapG in tumor cell dissemination and metastasis [14].

In this study, we used anti-CapG nanobodies as a tool to question whether the actin-binding properties of CapG are redundant at the protein level in a breast cancer metastasis model. Nanobodies are the smallest antigen-binding fragments from Camelid heavy-chain antibodies [17]. They are easily cloned and can be used as intrabodies. Importantly, they afford the opportunity to block selected biologic functions of a resident target antigen [18-20]. Our findings show that nanobody-based protein-directed CapG inhibition (immunomodulation) strongly reduces breast cancer metastasis, arguing against CapG redundancy. In view of the difficulties associated with developing small-compound inhibitors, we propose that a nanobody-based approach offers a fast track for gauging the role of distinct protein functions in cell physiology.

## Methods

MitoTracker Orange was purchased from Invitrogen (Merelbeke, Belgium) and used according to the manufacturer’s instructions. The pLV-CL vector was kindly provided by Sven Eyckerman (VIB Dept. Medical Protein Research, Ghent, Belgium). A polyclonal anti-CapG antibody was used as described earlier [10].

### Cloning

Nanobodies were generated as described [21]. In brief, a llama was injected subcutaneously on days 0, 7, 14, 21, 28, and 35 with 500 μg human recombinant CapG per injection. On day 39, anticoagulated blood was collected for preparation of lymphocytes. Total RNA from peripheral blood lymphocytes was used as template for oligo dT-primed first-strand cDNA synthesis. The VHH encoding sequences were amplified with PCR and cloned into the PstI and NotI sites of the phagemid vector pHEN4. A VHH library of about 10^7^ independent transformants was obtained. Three consecutive rounds of panning were performed on solid-phase coated CapG (10 μg/well). Enrichment for antigen-specific phages was assessed after each round of panning. The enrichment for antigen-specific phages was further evaluated by polyclonal phage ELISA.

Nanobodies were cloned in the pcDNA3.1/V5-His_6_-TOPO vector (Invitrogen) and its derivative (pMOM), in pEGFP-N1, and in pHEN6c [19,20] with a V5-tag. A nuclear export sequence (NES) from MAPKK or a nuclear localization sequence (NLS) from SV40 was additionally cloned in the BsrGI-site of pEGFP-N1. Plasmids pT3sCAPNb-Bla (Tc^R^) were constructed by substitution of the GFPNb sequence in pT3sGFPNb-Bla [22].

### Protein purification

Full-length wild-type human CapG and deletion fragments were subcloned in the pGEX-5X-1 vector. Recombinant proteins were produced in *Escherichia coli* TOP10 cells as GST-fusion proteins. For purification of untagged CapG, fusion proteins were cleaved with factor X_a_. Purification of recombinant V5-/His_6_-tagged nanobodies was performed as described previously [20].

### GST pull-down assay

Pull-down of CapG nanobodies with full-length or truncated GST-CapG was performed as described previously [19]_._

### Actin-binding assays

The actin monomer-binding assay was performed as described earlier [20]. *N*-(1- *pyrene*)iodoacetamide (pyrene)-actin (100 n*M*) (Cytoskeleton, Denver, CO, USA) was incubated with CapG (100 n*M*) in the absence or presence of recombinant CapG nanobodies (200 n*M*). Fluorescence was measured at 388 nm after excitation at 365 nm. In the high-speed sedimentation assay, 6 μ*M* actin (Cytoskeleton) was incubated with CapG (7,2 μ*M*) in the absence or presence of CapG nanobodies (14.4 μ*M*) in 1× G-buffer [20]. After addition of polymerization buffer [19], the reaction mixture was incubated for 30 minutes at room temperature. Samples were centrifuged for 30 minutes at 100,000 *g* to sediment F-actin. Pellet and supernatant fractions were separated by SDS-PAGE. Coomassie-stained protein bands were scanned, and densities were quantified with ImageJ. The actin-capping assay was performed essentially as described earlier [23] by using 7-chloro-4-nitrobenzeno-2-oxa-1,3-diazole (NBD)-labeled actin. Fluorescence was measured at 535 nm after excitation at 465 nm. Nanobodies were used at a concentration of 500 n*M*.

### Isothermal titration calorimetry

Binding of CapG to CapG nanobodies was measured at 30°C with ITC by using a Microcal VP-ITC, as described earlier [20]. Untagged CapG and His_6_-tagged CapG nanobodies were dialyzed against 20 m*M* Hepes, 150 m*M* NaCl, pH 7.5, in the presence of 50 μ*M* CaCl_2_ or 0.1 m*M* EGTA.

### Injection of nanobodies into MDA-MB-231 cells by attenuated EPEC carrying T3SS

Growth of attenuated enteropathogenic *E. coli* (EPEC) strains quad and Δ*escN*, infection of *in vitro* cultured human cell line MDA-MB-231, and the β-lactamase translocation assay were performed by following the conditions described previously [22].

### Stable cell lines

MDA-MB-231 cells stably expressing GFPNb- or GFP-tagged CapG nanobodies were created according to the manufacturer’s instructions (Lenti-XTet-On Advanced Inducible Expression System, Clontech, Saint-Germain-en-Laye, France). Nanobodies were cloned in the pLVX-Tight-Puro vector. For bioluminescent imaging, these cells were additionally retrovirally infected with the luciferase reporter (pLV-CL). Relative expression levels of CAPNbs (GFP-tagged) and endogenous CapG were calculated by using purified recombinant CapG and GFP as internal standards in immunoblot experiments.

### Breast tumor metastasis in mice

All animal work was performed in compliance with the guidelines of the ethical committee of the Faculty of Veterinary Medicine (Ghent University, EC2012/028). Llama immunization (Eurogentec, Seraing, Belgium) was performed and monitored by an accredited veterinarian. The animals were treated according to local guidelines for animal care and handling. Female 3– to 4-weeks-old immunodeficient NOD-SCID mice were used (Harlan Laboratories, Indianapolis, IN, USA). Mice received doxycycline (1 mg/ml) in 1% sucrose via their drinking water 4 days before injection of the cells and during the experiment. MDA-MB-231 cells stably expressing GFP or GFP-CAPNb2 and luciferase reporter were trypsinized and washed with PBS. The breast cancer lung-metastasis model was established by lateral tail vein injection of 1 × 10^6^ cells in 50 μl PBS. For the mammary fat pad tumor model, 2 × 10^6^ cells in 100 μl PBS containing 50% Matrigel were injected into the mammary fat pads (MFPs) of mice. One week before injection of cells in the MFPs, 3-weeks-old mice were subcutaneously injected with 1 mg estradiol in 100 μl sesame oil (Sigma, Diegem, Belgium). Primary tumor growth and development of metastases were monitored weekly after i.p. injection of 150 mg/kg D-luciferin by using an IVIS Lumina II System (Caliper Life Sciences, Teralfene, Belgium). From the time tumors were palpable, tumor length (*L*) and width (*W*) also were measured with a caliper. Tumor volume was calculated as *πLW*^2^/6.

At day 56, lungs were retrieved from mice and fixed in 10% formalin solution (Sigma) overnight. Tissues were embedded in paraffin, sectioned, stained with H/E, and analyzed with microscopy (Leica M205 FA20 Stereomicroscope).

### Immunoprecipitation, immunostaining, and microscopy

These assays were essentially performed as described earlier [19].

### Proteomics and mass spectrometry

See Additional file 1.

### Cell-cycle experiments

MDA-MB-231 stable cell lines were induced with doxycycline 48 hours before the experiment. Cells were detached by using 3 m*M* EDTA in PBS. After centrifugation for 10 minutes at 800 rpm, cells were fixed in 0.5% PFA for 1 hour at 4°C. Subsequently, cells were permeabilized in 70% ethanol overnight at 4°C. Finally, cells were resuspended in PBS with propidium iodide (40 μg/ml) and RNAse (100 μg/ml). Then 10,000 cells were analyzed with a FACS Calibur Flow Cytometer (CellQuest Pro software, BD Biosciences).

### Oris cell migration and invasion assay

Plates were coated with collagen (rat tail, type I, BD, 50 μg/ml, migration) or basement membrane extract (BME) solution (Matrigel; BD, 3.5 mg/ml, invasion) before seeding of the cells. Cells were seeded at 40,000 cells/well (in medium with 1% FBS and 1 n*M* EGF), except in the central well area, and allowed to attach for 3 hours. For migration experiments, images were acquired, without mask (CellM, IX81 Olympus fluorescence microscope). The area (μm^2^) covered by the cells was measured at several time points (maximum 36 hours) for the different replicates per condition. Migration efficiency is based on the mean slope of linearly fitting the area-over-time data (μm^2^/min). For the invasion assay, an additional layer of BME was applied on top of the cells, and fluorescence was measured with mask by using a FLX 800 microplate fluorescence reader (BIO-TEK instruments, INC).

### Statistical analysis

Data are expressed as means and standard deviation. For migration/invasion experiments and imaging data of mice, the SEM was used and analyzed with the Student *t* test, or a nonparametric test (ANOVA on ranks) was performed.

## Results

### CAPNb2 interaction with the first CapG domain inhibits G-actin binding and F-actin capping

**Figure 1:**
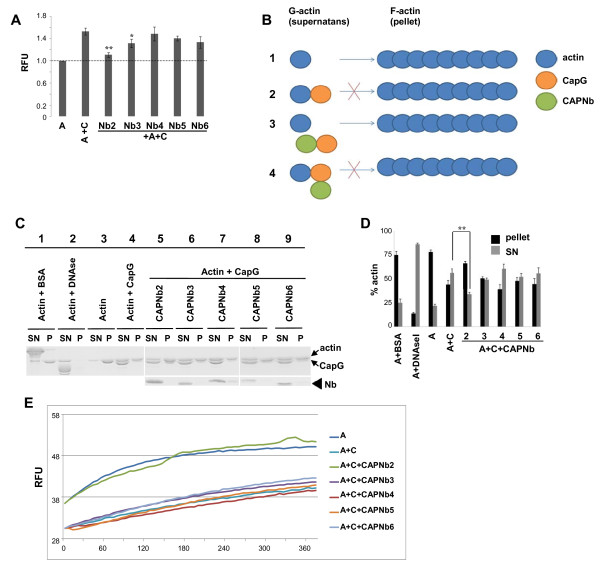
**CAPNb2 blocks CapG interaction with monomeric actin and actin filaments. (A)** G-actin-binding assay. CAPNb2 and 3 counteract a CapG-triggered increase in pyrene-labeled actin fluorescence. Intrinsic fluorescence of pyrene-actin was normalized to 1. A, actin; C, CapG; RFUs, relative fluorescence units. **(B)** Principle of the actin sedimentation assay. Actin monomers freely polymerize in the absence of CapG (1). CapG sequesters actin monomers, preventing their incorporation into filaments (2). If a nanobody prevents interaction between CapG and actin monomers, the sequestration effect is lost, and actin will polymerize (3). A nanobody may interact with CapG without affecting the sequestration of actin monomers, resulting in decreased polymerization (4). **(C)** CAPNb2 reduces the ability of CapG to sequester actin monomers in a sedimentation assay. Supernatants (SNs) and actin pellets (Ps) were fractionated with high-speed centrifugation. Proteins were separated with SDS-PAGE and visualized with Coomassie staining. Lanes 1, 2: controls. BSA does not affect actin polymerization. DNaseI (nanomolar affinity for G-actin) strongly blocks actin polymerization. Lanes 3/, –4: CapG reduces actin sedimentation. Actin level in the pellet is reduced while actin in the SN is increased. Lane 5: CAPNb2 counteracts the effect of CapG by increasing the actin fraction in the pellet and decreasing actin in the SN. Lanes 6 through 9: Other nanobodies have no significant effect. **(D)** Quantification of the experiment in **(C)**. A, actin; C, CapG. **(E)** F-actin-capping assay. Capping of F-actin seeds by CapG prevents the initial increase in fluorescence in the control condition and an increase in final F-actin level. The initial increase is due to high elongation rates of added F-actin seeds. CAPNb2 prevents CapG-dependent F-actin capping and results in actin polymerization kinetics comparable to the control. Student *t* test was performed: *P < 0.05; **P < 0.005; *n* = 3.

After immunization of a llama with human recombinant CapG, several CapG nanobodies (CAPNb) were obtained (see Materials and Methods). Because CapG regulates cytoskeletal organization through Ca^2+^-dependent interaction with globular (G-) actin and filamentous (F-) actin [8], we screened for nanobodies that perturb these properties. For brevity, results are shown for selected nanobody classes (based on the sequence of their third complementarity-determining region (CDR3)). In Western blot experiments, several nanobodies belonging to different classes specifically recognized a ~40-kDa protein, corresponding to CapG, present in protein extracts obtained from MDA-MB-231 breast cancer cells (see Additional file 1: Figure S1). To investigate G-actin binding, we performed a fluorescence-based assay using pyrene (*N*-(1-*pyrene*)iodoacetamide)-labeled actin. The fluorescence intensity of pyrene-conjugated actin increases on CapG binding [24] (Figure 1A). This increase in fluorescence is modest because, unlike gelsolin, CapG binds to only one actin monomer under nonpolymerizing conditions [24]. In the presence of CAPNb2 or CAPNb3, the fluorescence decreased significantly, indicating that these nanobodies (partially) prevent CapG interaction with G-actin. CAPNb4, 5, and 6 showed no significant effect.

We next performed F-actin sedimentation assays by incubating actin with an equimolar concentration of CapG under conditions that favor actin polymerization (Figure 1B,C). Actin filaments and monomeric actin were separated with high-speed centrifugation, and the resulting pellets and supernatants were analyzed with SDS-PAGE and quantified. The extent of polymerization (that is, actin in pellet) is reduced as a result of G-actin scavenging by CapG (Figure 1B,C, lane 4), unless a nanobody blocks CapG-actin interaction. After incubation with CAPNb4, the bulk of actin was found in the supernatant with a smaller F-actin fraction in the pellet, suggesting that CAPNb4 has no effect on actin binding by CapG (Figure 1C, lane 7). However, we noticed a reversal in the G-/F-actin distribution on addition of CAPNb2. More actin was recovered in the pellet (Figure 1C, lane 5). Quantification of these experiments (Figure 1D) demonstrates that only CAPNb2 significantly inhibits actin monomer binding by CapG.

We then determined whether nanobodies inhibited F-actin capping by CapG. The fluorescence of short unlabeled F-actin nuclei increased when 7-chloro-4-nitrobenzeno-2-oxa-1,3-diazole (NBD)-labeled actin monomers were added, but this was inhibited by CapG (Figure 1E). The filaments remained capped in the presence of all nanobodies that were tested, except for CAPNb2. CAPNb2 blocked capping, as evidenced by the reappearance of polymerization kinetics similar to the conditions in which NBD-actin was polymerized alone (Figure 1E). Thus, from the set studied, CAPNb2 was the only nanobody inhibiting the association of CapG with both G-actin and F-actin. For this reason, we selected this nanobody for further detailed analysis, by using other CAPNbs as controls.

Calorimetry experiments (thermodynamic parameters are summarized in Additional file 1: Table S1) revealed that the *K*_d_ of CAPNbs for CapG ranged from 150 n*M* to 0.8 n*M* in the presence of calcium. Interaction is mostly enthalpy driven (Additional file 1: Figure S2). CAPNb2 (*K*_d_ = 23 n*M* and 4 (*K*_d_ = 5.3 n*M*) interact with CapG in a Ca^2+^-dependent manner (Additional file 1: Table S1), unlike CAPNb7 which binds to CapG irrespective of calcium, suggesting an interaction with different epitopes.

**Figure 2:**
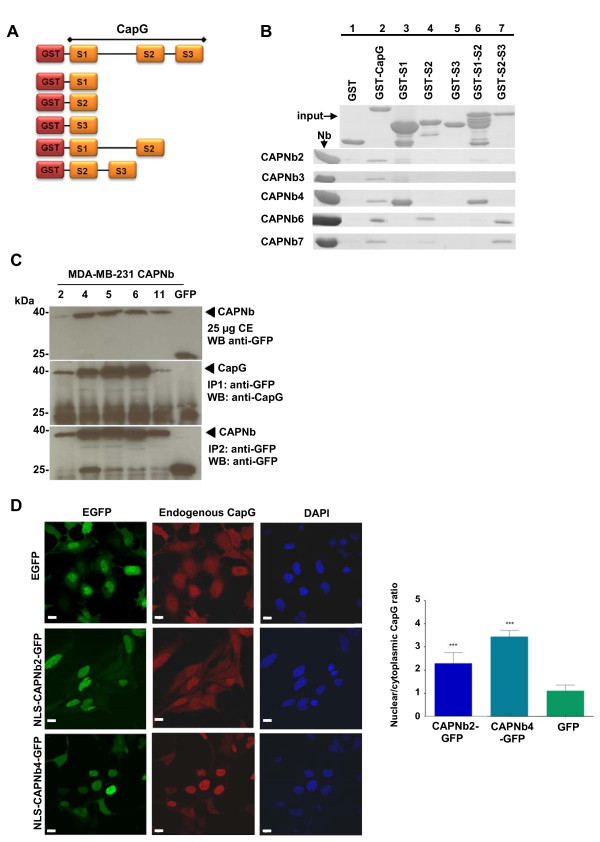
**Epitope mapping and nanobody functionality as intrabody. (A)** Schematic representation of GST-fusion proteins used in pull-down experiments. **(B)** Epitope mapping. Glutathione-*S*-transferase pull-down assays with recombinant CAPNbs (left, input) and GST-CapG fragments (indicated on top) were evaluated with SDS-PAGE and coomassie staining. **(C)** Co-immunoprecipitation. EGFP-tagged CAPNbs or a GFP control (numbers indicated on top) were stably expressed in MDA-MB-231 cells and detected in cell extracts (top panel). They were retrieved from cell extracts with a polyclonal anti-EGFP antibody (bottom panel). All CAPNbs immunoprecipitated endogenous CapG (middle panel). CE, crude extract. **(D)** Upper panels: MDA-MB-231 cells were transfected with EGFP (control) and stained for endogenous CapG (red) and show the typical nucleocytoplasmic distribution of CapG. Middle panels: MDA-MB-231 cells were transfected with CAPNb2-EGFP coupled to the SV40 large T-antigen nuclear-localization sequence (NLS), promoting a stronger CapG enrichment in the nucleus. Lower panels: MDA-MB-231 cells were transfected with CAPNb4-EGFP coupled to the SV40 large T-antigen NLS, redirecting endogenous CapG (red) nearly completely to the nucleus. DAPI staining of the nucleus is shown in blue at right. Bar, 10 μm. Quantification of this experiment is shown in the histogram at right. The nuclear enrichment of CapG was evaluated by determining the ratio of cytoplasmic versus nuclear CapG. Statistics were performed on 50 cells per condition, with the EGFP (control) condition as reference (***P < 0.001).

To address this further, we used glutathione-*S*-transferase (GST) pull-down experiments. CapG is a member of the gelsolin family of actin-associated proteins. This family is characterized by the presence of three or six structural repeats of ~120 amino acids. Full-length CapG and truncation mutants were used, consisting of a single gelsolin-like repeat (S1, S2, or S3) or a combination of two repeats (Figure 2A,B, top panel). As shown in Figure 2B, all nanobodies were retained by full-length CapG (lane 2) but did not react to a significant degree with GST alone (Figure 2B, lane 1). Although some deletion fragments of CapG were subject to partial degradation, we observed that CAPNb4 binds strongly to GST-S1 and GST-S1-S2, suggesting that its epitope is located in the S1 domain. The same applies to CAPNb2 and 3, although their interaction was overall weaker compared with CAPNb4 or below the detection limit (CAPNb3 and GST-S1-S2). This may be caused by the bulky GST moiety, which is twice the size of a nanobody, and GST could interfere with nanobody binding to CapG (note that ITC experiments were performed with untagged CapG).

Alternatively, CapG deletion fragments may lack part of an epitope. For instance, linker regions between the CapG domains may also constitute part of the epitope recognized by nanobodies. Nanobodies 6 and 7 interact with GST-S2-S3 and GST-S2, indicating that they, at minimum, bind to the second CapG domain. Thus CAPNbs target different epitopes in CapG.

We expressed EGFP-tagged nanobody cDNAs in MDA-MB-231 breast cancer cells by lentiviral transduction to verify their ability to act as *bona fide* CapG binders in the cytoplasmic environment (as intrabodies). GFP-expressing or parental MDA-MB-231 cells were used as negative control. CapG nanobodies were able to co-immunoprecipitate CapG (Figure 2C; Additional file 1: Figure S3A), indicating that they recognize endogenous CapG. To substantiate this further, we performed delocalization experiments designed to alter intentionally the subcellular distribution of endogenous CapG. CapG is known to shuttle between the nucleus and cytoplasm [25,26] and to distribute evenly between both compartments (Figure 2D, top panel). Tagging of CAPNb2 with the nuclear-localization sequence (NLS) of SV40 large T-antigen promoted enrichment of endogenous CapG in the nuclear compartment (Figure 2D, middle panel), and this effect was even more pronounced for NLS-CAPNb4 (Figure 2D, lower panel). The apparent weaker activity of CAPNb2 compared with CAPNb4 may be attributed to its lower affinity (Additional file 1: Table S1) and the higher expression level of CAPNb4 (Figure 2C and Additional file 1: Figure S5A). Similar findings were obtained with additional targeting strategies (Additional file 1: Figure S3B-K). We therefore conclude that nanobodies interact with CapG in the cytoplasm as well as in distinct cell compartments.

### CAPNb2 induces multinucleation

To advance our understanding of the CAPNb2 mode of action, we used quantitative proteomics involving SILAC to label proteomes of MDA-MB-231 cells differentially (Additional file 1: Figure S4A). By using LC-MS/MS analysis, we compared the CAPNb2 and CAPNb4 interactomes (the complete protein list is shown in Additional file 1: Figure S4C). We found that different tubulin isoforms (tubulin beta chain and beta-4B chain) were enriched in the CAPNb2 interactome (Additional file 1: Figure S4B, Additional file 2 for complete list).

Although the biologic implications are not yet understood, and further analysis is required, it was suggested previously that CapG mediates cross-talk between the actin cytoskeleton and microtubule-based organelles that regulate cell division. CapG localizes at the mother centriole in interphase, the mitotic spindle in mitosis, and the midbody ring in abscission [27]. Furthermore, genome-wide transcript profiling of the cell cycle also revealed upregulation of CapG in G_2_, before the onset of mitosis [28].

**Figure 3:**
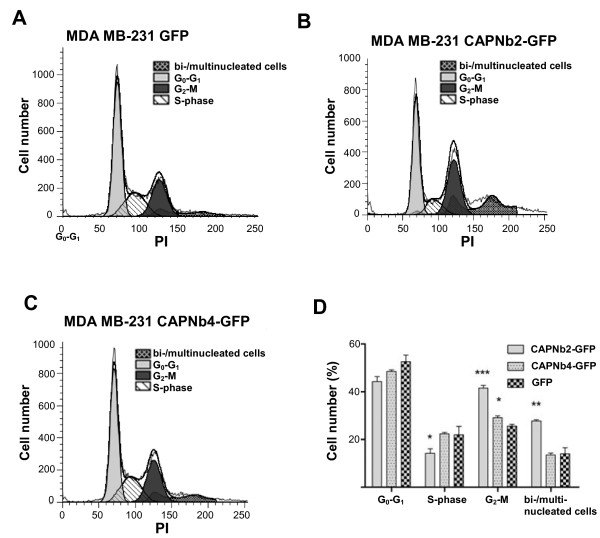
**CAPNb2 induces moderate multinucleation.** Flow-cytometry profiles. Unlike MDA-MB-231-GFP **(A)** or MDA-MB-231-CAPNb4 cells **(C)**, MDA-MB-231-CAPNb2 cells **(B)** harbor a larger proportion of G_2_/M cells (*t* test, *P* = 0.0003, *n* = 3) and bi/multinucleated cells (*t* test, *P* = 0.006; *n* = 3). Cells in S-phase are significantly decreased (*t* test, *P* = 0.01; *n* = 3). A CAPNb4-triggered increase in G_2_/M cells is modest but significant (*t* test, *P* = 0.04; *n* = 3). PI, propidium iodide. **(D)** Histogram summarizing the data shown in (**A** through **C**), including statistical analysis (comparison with GFP-expressing control cells). (*p<0.05, **p<0.01 and ***p<0.001).

As these data suggest that CAPNb2 might affect cell division, we analyzed MDA-MB-231-CAPNb cells with flow cytometry. Results showed that CAPNb2 gives rise to a significant increase in G2/M cells (compared to GFP control cells) as well as to a third cell subpopulation (Figure 3B,D), indicative of multinucleated cells, compared with MDA-MB-231 cells expressing either GFP (Figure 3A,D) or CAPNb4 (Figure 3C,D). These changes occur primarily at the expense of cells in S-phase and, to a minor extent, a reduction in the G_0_/G_1_ population.

### CAPNb2 restricts migration, Matrigel invasion, and metastasis of MDA-MB-231 breast cancer cells

**Figure 4:**
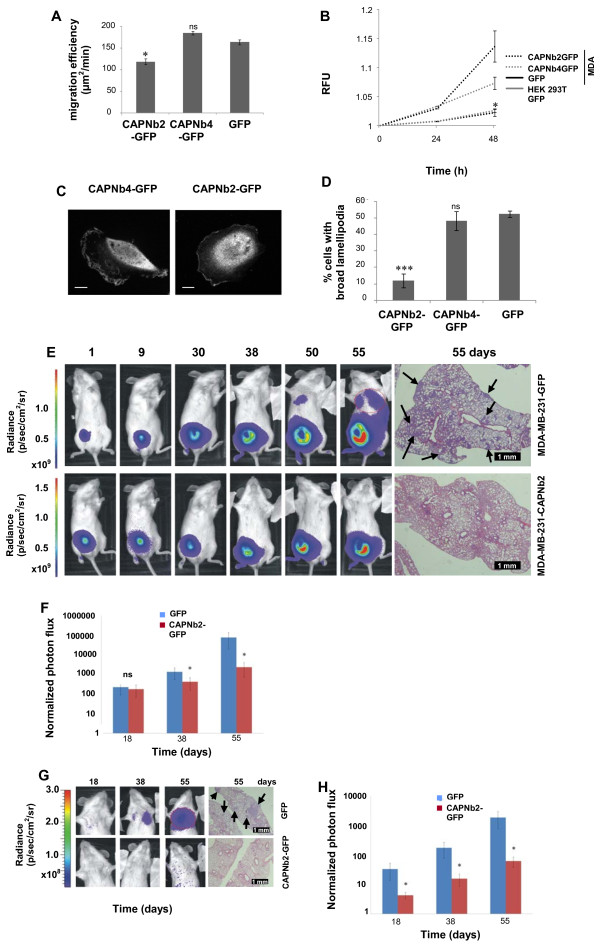
**CAPNb2 restrains lung metastasis of breast cancer cells. (A)***In vitro* cell migration. CAPNb2 significantly reduces migration of MDA-MB 231 cells; *n* = 6 (CAPNb2), 7 (CAPNb4) and 7 (GFP), *P* = 0.017 CAPNb2 versus GFP. Ns, not significant. **(B)** Matrigel invasion. MDA-MB-231 cells stably expressing CAPNb2 are not invasive compared with GFP-transduced cells (*t* test, *P* = 0.046; *n* = 3). RFU, relative fluorescence unit. **(C, D)** MDA-MB-231-CAPNb2-EGFP cells display fewer broad lamellipodia (*t* test, *P* = 0.00013; *n* = 3). **(E)** Orthotopic metastasis model. Noninvasive bioluminescence images of mice injected with GFP control cells or CAPNb2 cells. A red line (55d) marks the region of interest. H/E stainings of lung sections are at right. Arrows, macrometastases. **(F)** Normalized photon-flux profile from lungs at the indicated times in the orthotopic metastasis model. t_38_*P* = 0.0240; t_55_*P* = 0.0339; *n* = 6 to 7. **(G)** Tail-vein metastasis model. Bioluminescence images of mice injected with GFP control cells or CAPNb2 cells. A red line (55d) marks the region of interest. H/E stainings of lung sections are at right. Arrows, macrometastases. **(H)** Normalized photon-flux profile from lungs at the indicated times in the tail-vein metastasis model. t_18_*P* = 0.0204; t_38_*P* = 0.0092; t_55_*P* = 0.0104; *n* = 7. A mouse in the GFP cohort (*n* = 4) with an unusually high normalized photon flux of 2.93 × 10^7^ photons/s was excluded. The total photons/s was measured in a standard-sized circular region of interest encompassing the murine chest. For normalization, we set to 1 the average photon flux at day 0 of the lungs of mice injected with CAPNb2-GFP cells. Error bars represent mean ± SEM except for D, in which mean ± SD are shown. *P < 0.05; ***P < 0.001.

To ascertain whether CAPNb2 reduces the migratory and invasive properties of cells *in vitro*, we used ORIS migration and invasion assays [29]. We used MDA-MB-231 cells with doxycycline-inducible expression of GFP-tagged nanobodies (Additional file 1: Figure S5A). By using recombinant CapG and GFP as internal standards, we calculated that CAPNb2 is expressed at a concentration equivalent to endogenous CapG (Additional file 1: Figure S5B). CAPNb4 expression is 4.4-fold higher (Additional file 1: Figure S5A). As depicted in Figure 4A, CAPNb2-EGFP expressing MDA-MB-231 cells showed markedly reduced migration capabilities on a 2D collagen coating, as compared with control (GFP) or CAPNb4-EGFP-expressing cells. Changing the growth conditions from 10% FBS to 1% FBS/1 n*M* EGF demonstrated that the three cell lines had comparable S-phase populations, indicating that proliferation effects can be excluded (Additional file 1: Figure S5C). Next, Matrigel invasion was investigated by using the ORIS invasion system. CAPNb2-expressing cells lost their capacity to invade the Matrigel, whereas CAPNb4 triggered only a partial reduction in invasion of MDA-MB-231 cells (Figure 4B). Microscopic analysis showed a less-polarized morphology of CAPNb2-expressing cells with fewer broad lamellipodia (containing membrane ruffles), as compared with CAPNb4 or GFP-MDA-MB-231 control cells (Figure 4C,D).

**Figure 5:**
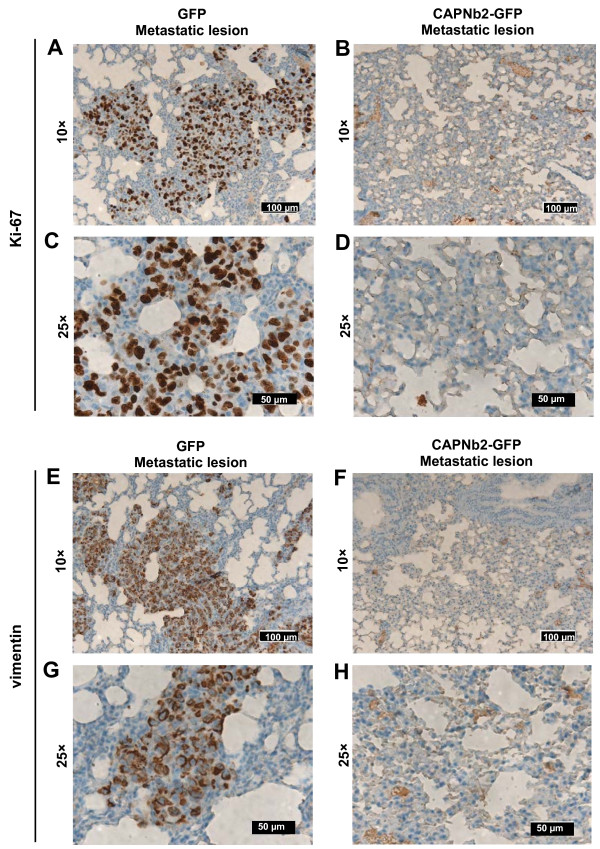
**Ki-67 and vimentin staining of lung metastatic lesions. (A-D)** Ki-67 staining of histologic sections. Representative images showing proliferating cells in lung sections obtained from animals injected with MDA-MB-231-GFP cells **(A,C)** or MDA-MB-231-CAPNb2 cells **(B,D)**. Erythrocyte peroxidase activity contributes to the weak staining in **(A,D)**. **(E-H)** Vimentin staining. Representative images showing vimentin-positive MDA-MB-231 cells in lung sections obtained from mice injected with MDA-MB-231-GFP cells **(E,G)** or MDA-MB-231-CAPNb2 cells **(F,H)**.

We next determined whether CAPNb2 could prevent metastasis of breast cancer cells in mice. MDA-MB-231-CAPNb2-GFP and MDA-MB-231-GFP (control) cells were transduced with a luciferase reporter (Additional file 1: Figure S5D) and injected into the mammary fat pads or into the tail vein of immunodeficient (NOD-SCID) mice. Subsequently, the occurrence of lung metastases was monitored with noninvasive bioluminescence imaging. CAPNb2 markedly restrained the occurrence of lung metastases in both the orthotopic (Figure 4E,F) and tail-vein (Figure 4G-H) models (>90% reduction in photon flux). Postmortem examination of the animals confirmed that bioluminescence in the lungs originated from metastasized cells (data not shown). Hematoxylin and eosin staining of lung sections revealed many macrometastases in animals injected with GFP control cells (Figure 4E,G right, upper panels). Ki-67 immunostaining indicated highly proliferative potential of GFP cells (Figure 5A,C) and macrometastatic colonies stained positive for human vimentin (Figure 5E,G). In contrast, mice injected with MDA-MB-231-CAPNb2 cells showed no macrometastases in the lungs; few micrometastatic lesions were observed at higher magnification (Figure 5B,D,F,H). Hematoxylin and eosin staining of lung metastatic lesions in tail vein and orthotopic models, as well as Ki-67/vimentin staining of primary tumor sections, are shown in Additional file 1: Figure S6 and S7, respectively. Interestingly, data suggest that CAPNb2 also affected primary tumor growth and local invasion into the vascular layers of the abdominal wall. The average tumor volume after 8 weeks was approximately 3 times smaller than the tumor volume of mice injected with control cells (Additional file 1: Figure S5E).

### Injection of CAPNbs into MDA-MB-231 cells by the type III protein-secretion system (T3SS) of *E. coli*

**Figure 6:**
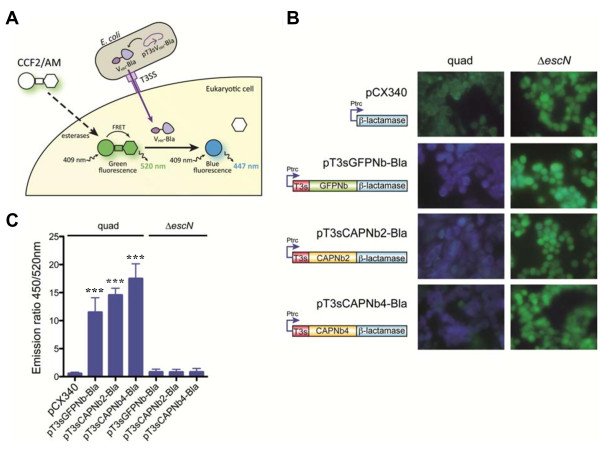
**T3SS-mediated injection of CapG nanobodies in MDA-MB-231 cells. (A)** Schematic depicting injection of a VHH (nanobody) fused to a T3-secretion signal (T3s) and β-lactamase (Bla) reporter into a mammalian cell by the T3SS of enteropathogenic *E. coli* (EPEC) strains. The nonfluorescent Bla substrate (CCF2/AM) is transformed by cellular esterases to the green fluorescent substrate CCF2. CCF2 hydrolysis by Bla results in blue fluorescence. **(B)** Blue fluorescence is observed on infection with attenuated EPEC bacteria (quad) carrying T3s-Bla fusions of GFPNb (positive control), CAPNb2 and CAPNb4. Green fluorescence is observed in MDA-MB-231 cells infected with ∆*escN* strains (lacking the essential ATPase of the T3SS) or a Bla construct lacking the T3s signal sequence (pCX340). **(C)** Quantification of the fluorescence intensity ratio 447/520 nm of cultures of MDA-MB-231 cells infected with attenuated EPEC quad strains, or EscN-deficient strains, carrying pCX340 (negative control) and T3s-Bla fusions of GFPNb, CAPNb2, and CAPNb4. Results are expressed as mean ± SEM of three independent infection experiments for every bacterial strain. Data were analyzed by using one-way analysis of variance (ANOVA) and Bonferroni Multiple Comparison Test with the PRISM software (Graphpad) to calculate the *P* values of each data set with the control (quad pCX340). Asterisks (***) indicate *P* < 0.001.

Unmodified nanobodies do not spontaneously cross the plasma membrane (our unpublished data), a constraint limiting their therapeutic potential when targeting intracellular proteins. Some enteropathogenic *E. coli* (EPEC) use a type III protein-secretion system (T3SS) to inject effector proteins into cells [30-32]. The T3SS of EPEC strains was shown to inject a GFP-specific nanobody (GFPNb) into the cytoplasm of HeLa cells [22]. As with GFPNb, we tagged CapG nanobodies with the N-terminal T3s signal sequence of the *E. coli*-secreted protein F (EspF) effector and the β-lactamase (Bla) reporter (Figure 6A) [22,33]. Plasmids were transformed into an attenuated EPEC strain assembling a functional T3SS, named quad [22]. This strain is a quadruple mutant of EPEC wild-type strain E2348/69 [34] that lacks the adhesin Intimin and three major effectors (Tir, EspF, and Map) [35,36]. Plasmids were also transformed into an EPEC E2348/69 ∆*escN* mutant lacking the essential ATPase of the T3SS [22,36]. MDA-MB-231 cells were infected with these bacteria and incubated with the Bla substrate CCF2. Hydrolysis of CCF2 by translocated nanobodies fused to Bla caused a shift in its fluorescence emission from 520 nm (green) to 450 nm (blue) (Figure 6A,B). Microscopic analysis demonstrated that GFPNb, CAPNb2 and 4 are injected into the cytoplasm of MDA-MB-231 cells by the attenuated EPEC quad bacteria (Figure 6B, left panels). In contrast, green fluorescence was observed in MDA-MB-231 cells infected with ∆*escN* strain (Figure 6B, right panels). The fluorescence of infected MDA-MB-231 cells was quantified in three independent infection experiments, showing a clear increase in the blue/green 450/520-nm fluorescence ratio with EPEC quad bacteria carrying T3s-Bla fusions of GFP or CapG nanobodies (Figure 6C). Therefore, attenuated EPEC bacteria with a functional T3SS can inject CAPNbs into the cytoplasm of MDA-MB-231 cells.

## Discussion

Our genome encodes several proteins with actin filament-capping activity [7,37]. On the basis of this multiplicity, it has been proposed that similar proteins perform overlapping functions. Consequently, inactivation or genetic ablation of one capping protein is not expected to trigger major defects in cell physiology because similar proteins can perform the same task. CapG-knockout mice are viable, fertile, and show no gross pathologic defects, suggesting that CapG is redundant at the genetic level during development [6]. However, the findings reported here suggest that this may not necessarily hold true at the protein level or under pathologic conditions, because CAPNb2 reduced metastatic spread by >95% in orthotopic and tail-vein models of metastasis by preventing CapG from binding to actin filaments and regulating their polymerization status. Surprisingly, we did not observe gross changes in the organization of the actin cytoskeleton in MDA-MB-231 cells that express CAPNb2. However, similar findings were reported previously in cells that overexpress CapG [9]. Although MDA-MB-231 cells are known to express substantial amounts of other capping proteins, such as twinfilin and gelsolin, these polypeptides cannot act as a CapG proxy. As such, these findings point to an important role for CapG-dependent regulation of actin-filament turnover during metastasis, and this is supported by several independent proteomic studies [12,14-16,38]. Hypoxic conditions probably contribute to CapG-dependent enhanced metastasis. Several functional HIF-1α-responsive elements have been identified by reporter assays and chromatin immunoprecipitation in the region upstream of the *CAPG* gene. Furthermore, HIF-1α or hypoxic conditions enhance CapG expression in leukemic U937T cells [39].

Based on our results, we speculate that many other structural proteins may be endowed with a higher therapeutic potential than anticipated at present. Frequently, however, specific and high-affinity inhibitors targeting such proteins are wanting, because of their non-enzymatic character. Macroketones that target the actin-bundling protein fascin [40] or the Arp2/3 inhibitors CK-0944636 and CK-0993548 [41] are an exception in this regard. Unlike these small compounds, however, expression of an intrabody can be tuned in an inducible manner and adjusted to the expression level of the target antigen, thereby minimizing off-target effects.

In addition, when applied to multidomain or multifunctional proteins, nanobodies may provide more possibilities for selective functional inhibition. Nanobodies may thus progress into a powerful instrument to help explain the myriad phenomena that are based on the cellular actin system, and which require new tools and models [42]. Whether CapG represents a *bona fide* therapeutic target in relation to metastasis requires further investigation as well as expansion of currently available technologies. Nevertheless, we argue that nanobodies in general appropriately mimic the activity of pharmacologic compounds by directly targeting a protein (function) of interest. Through detailed mapping of a therapeutic epitope, (that is, by x-ray crystallography [43]), nanobodies can be valuable as a stepping stone toward rational design of organic compounds that bind to such an epitope. Further elaboration of the nanobody-injection approach with nonpathogenic *E. coli* bacteria endowed with a functional T3SS will make a whole new repertoire of putative targets more accessible, many of which play a role in the etiology or progression of various diseases, including, but not limited to, cancer.

In this regard, it is interesting to note the capacity of commensal and probiotic *E. coli* to selectively proliferate in solid tumors, including breast tumors, after systemic administration [44,45] and that certain probiotic *E. coli* strains are currently used in human therapy [46,47].

## Conclusions

Perturbing the interaction between CapG and actin strongly reduces breast cancer metastasis in immunodeficient mice. Gene products that are redundant at the genetic level may yet represent valid drug targets. It is to be expected that protein-level inhibition of other cytoplasmic structural (nonenzymatic) polypeptides in this manner will contribute to quick assessment of their role in cancer cell behavior. Given their ease of production, in addition to high specificity/affinity and stability *in vitro* and *in vivo*, we argue that nanobodies represent a preferred instrument to trigger protein functional knockouts, thereby adequately simulating drug activity.

## Abbreviations

2D: Two-dimensional; Bla: β-lactamase; CAPNb: CapG nanobody; EPEC: Enteropathogenic *E. coli*; GFPNb: GFP nanobody; GST: Glutathione*-S-*transferase; Nb: Nanobody; TS33: Type III protein-secretion system.

## Competing interests

The authors declare that they have no competing interests.

## Authors’ contributions

JB, CB, OZ, EM, and BV characterized CapG nanobodies. KVI, SC, ODW, OZ, and NS performed mouse experiments. MVT and KVI designed and performed cell-migration experiments. GHG was involved in the immunization, library construction, and phage panning of nanobodies. KL carried out immunohistochemistry. DRG and LAF designed T3SS experiments. FI and KG carried out proteomics experiments. JVDK participated in the design of the study. JG and KVI conceived the study and drafted the manuscript. All authors read and approved the final manuscript.

## Supplementary Material

Additional file 1: Table S1 Thermodynamic parameter summary of Isothermal Titration Calorimetry (ITC) measurements between nanobodies and CapG. **Figure S1.** Different CAPNb classes recognize endogenous CapG. **Figure S2.** Gibbs free energy, enthalpy, and entropy changes associated with CapG-CAPNb interaction calculated from ITC measurements. **Figure S3.** Validation of CapG nanobodies *in vitro* and *in vivo*. **Figure S4.** Quantitative proteomics profiling. **Figure S5.** Inducible CAPNb expression in breast cancer cells. **Figure S6.** Histologic sections of lung metastatic lesions. **Figure S7.** Ki-67 and vimentin staining of primary tumor sections. Click here for file

Additional file 2 Proteins quantified and validated by Rover (proteomics). Click here for file
