# Supplementary material for: A nanobody targeting the F-actin capping protein CapG restrains breast cancer metastasis
Source: Breast Cancer Res. 2013 Dec 13;15(6):R116. doi: 10.1186/bcr3585 (PMC3979033; doi:10.1186/bcr3585)
Supplement: Additional file 1: Table S1 — Thermodynamic parameter summary of Isothermal Titration Calorimetry (ITC) measurements between nanobodies and CapG. Figure S1. Different CAPNb classes recognize endogenous CapG. Figure S2. Gibbs free energy, enthalpy, and entropy changes associated with CapG-CAPNb interaction calculated from ITC measurements. Figure S3. Validation of CapG nanobodies in vitro and in vivo. Figure S4. Quantitative proteomics profiling. Figure S5. Inducible CAPNb expression in breast cancer cells. Figure S6. Histologic sections of lung metastatic lesions. Figure S7. Ki-67 and vimentin staining of primary tumor sections. [file bcr3585-S1.docx]

**Additional file 1**

**A nanobody targeting the F-actin capping protein CapG restrains breast cancer metastasis.**

Katrien Van Impe^1,9^, Jonas Bethuyne^1,9^, Steven Cool^2^, Francis Impens^1,3^, David Ruano-Gallego^4^, Olivier De Wever^5^, Berlinda Vanloo^1,3^, Marleen Van Troys^1^, Kathleen Lambein^6^, Ciska Boucherie^1^, Evelien Martens^1^, Olivier Zwaenepoel^1^, Gholamreza Hassanzadeh-Ghassabeh^7^, Joël Vandekerckhove^1^, Kris Gevaert^1,3^, Luis Ángel Fernández^4^, Niek N. Sanders^2^ and Jan Gettemans^1,8^. ^9^These authors contributed equally to this work.

**Supplementary methods**

**Proteomics**

For SILAC labelling, MDA-MB-231 cells were cultured in DMEM without L-Lysine, L-Arginine and L-Glutamine (Silantes Gmbh) supplemented with 10 % dialyzed foetal bovine serum (Invitrogen), 100 units/ml penicillin (Invitrogen), 100 µg/ml streptomycin (Invitrogen), 2 mM GlutaMAX (Invitrogen), ^12^C_6_ L-Lysine.HCl and ^12^C_6_ L-Arginine.HCl (L; light medium) or ^13^C_6_ L-Lysine.HCl and ^13^C_6_ L-Arginine.HCl (H; heavy medium) (Silantes Gmbh). While L-Lysine.HCl was added at its normal concentration in DMEM medium (146 mg/L), the concentration of L-Arginine.HCl was reduced to 21 mg/L (25% of the normal concentration in DMEM medium) to prevent metabolic arginine to proline conversion. Cells were kept at 37°C in 5% CO_2_ for at least six population doublings to ensure complete incorporation of the labelled lysine and arginine.

CapG was immunoprecipitated from lysates with recombinant V5-tagged CAPNb2 or CAPNb4, followed by incubation with anti-V5 agarose. Immunoprecipitation with CAPNb2 was performed in 1H and 4L labelled cultures, while CAPNb4 was used in 1L and 4H labelled cultures according to a previously described experimental design that avoids the presence of singleton peptides in MS spectra [1]. In this setup, CapG and proteins that interact independently of the presence of both nanobodies are expected at L/H ratios of 1/1, while proteins that interact specifically with CapG in the presence of CAPNb2 or CAPNb4 will show L/H ratios that deviate from 1/1 with a maximum of 4/1 or 1/4, respectively. Immunoprecipitations were performed separately for all 10 cultures after which eluates were mixed to retain one protein sample that was dried completely under vacuum.

**Mass spectrometry**

Proteins were re-dissolved in 500 µl 50 mM triethylammonium bicarbonate and digested overnight at 37˚C with 2 µg sequencing grade trypsin (Promega). The resulting peptide mixture was dried completely, re-dissolved in 100 µl 2% acetonitrile in 10 mM ammonium acetate pH 5.5 and separated by reversed phase HPLC. Peptides were collected in 1-min fractions and re-pooled with a 20 min time interval, dried completely and re-dissolved in 30 µl 2% acetonitrile in water to generate 20 samples for LC-MS/MS analysis on a Thermo LTQ Orbitrap XL mass spectrometer (2.5  *μ*l of sample was injected for each reading). In total, 19714 MS/MS spectra were recorded and examined using a locally installed version of the Mascot algorithm in the human subsection of the Swiss-Prot database (UniProtKB/Swiss-Prot database, release from March 8 2011 containing 20,233 human protein sequences) with additional entries for CAPNb2 and CAPNb4. The following search parameters were used: the protease setting was set to Trypsin/P with up to one missed cleavage allowed, variable acetate modification of peptide N-termini, variable oxidation of methionine residues to methionine-sulfoxide, variable formation of pyroglutamate from N-terminal glutamine residues, precursor mass tolerance of 10 ppm and fragment mass tolerance of 0.5 Da. To allow identification of peptides from both light and heavy labelled samples, a quantitation method was made defining a light and heavy component with ^12^C_6_ Arg, ^12^C_6_ Lys and ^13^C_6_ Arg, ^13^C_6_ Lys as exclusive modifications at any position, respectively. Only peptides that were ranked first and scored above the Mascot identity threshold at 99% confidence interval were withheld. Identified peptides were quantified using the Mascot Distiller Quantitation Toolbox in the ‘precursor’ mode as described before [1]. In total, 2725 fragmentation spectra were identified (with an FDR of 0.4 %) [2] and quantified peptides were visualized and manually validated for selected proteins using Rover [3]. For processing of all MS data, the ms_lims software platform was used [4].

**Table S1**. Thermodynamic parameter summary of Isothermal Titration Calorimetry (ITC) measurements between nanobodies and CapG.

|  | **Nb2** | | **Nb3** | **Nb4** | | **Nb5** | | **Nb6** | **Nb7** | | **Nb11** | **Nb13** |
| --- | --- | --- | --- | --- | --- | --- | --- | --- | --- | --- | --- | --- |
|  | -Ca^2+^ | +Ca^2+^ | +Ca^2+^ | -Ca^2+^ | +Ca^2+^ | -Ca^2+^ | +Ca^2+^ | +Ca^2+^ | -Ca^2+^ | +Ca^2+^ | +Ca^2+^ | +Ca^2+^ |
| **N*** | **** | 0.91 | 0.98 | **** | 0.74 | 0.98 | 0.91 | 0.87 | 0.91 | 1.05 | 0.95 | 1 |
| **ΔH**  **(kcal/mol)** |  | -27.5 | -19 |  | -11 | -21 | -21.1 | -15.5 | -5.6 | -5.2 | -5.5 | -20.1 |
| **ΔS**  **(cal/mol K)** |  | -55.9 | -31.3 |  | 1.48 | -28.8 | -27.9 | -11.8 | 16 | 17.7 | 17.5 | -28.4 |
| **TΔS****  **(kcal/mol)** |  | -16.9 | -9.5 |  | 0.4 | -8.7 | -8.5 | -3.6 | 4.9 | 5.4 | 5.3 | -8.6 |
| **ΔG*****  **(kcal/mol)** |  | -10.6 | -9.5 |  | -11.4 | -12.3 | -12.6 | -11.9 | -10.5 | -10.6 | -10.8 | -11.5 |
| **K_d_ (nM)** |  | 23 | 150 |  | 5.3 | 1.6 | 0.77 | 2.7 | 31 | 25 | 15 | 4.8 |

*N=stoichiometry

** with T (Kelvin) =303.15

***ΔG = ΔH-TΔS

****no significant binding


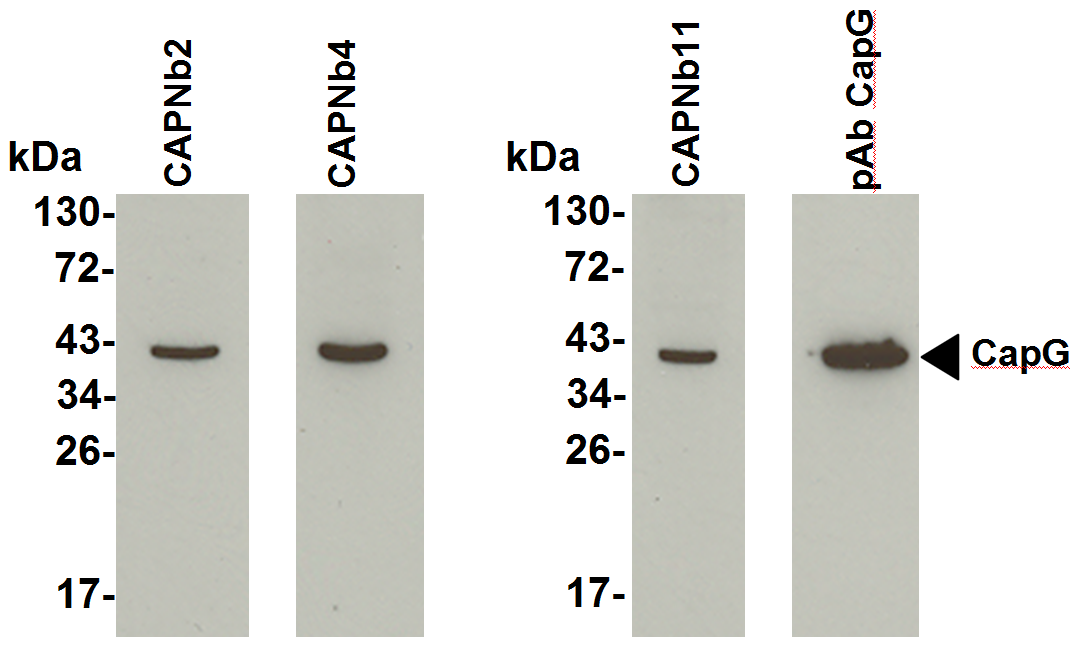


**Figure S1. Different CAPNb classes recognize endogenous CapG.**

MDA-MB-231 cells were lyzed in ice cold PBS with 0.5% NP-40, 1 mM PMSF and 1 mM protease inhibitor cocktail mix. 50µg protein extract, obtained after centrifugation at 20,000 ×g for 10 min, was fractionated by SDS-PAGE and blotted onto a nitrocellulose membrane. 4 µg of the respective V5-tagged nanobodies was used as primary antibody, followed by addition of a monoclonal anti-V5 antibody. Affinity-purified rabbit polyconal anti-CapG antibody [5-7] was diluted 1:2000. Proteins were visualized by enhanced chemiluminescence

detection (Amersham Pharmacia Biotech). Molecular weight markers are indicated.

**
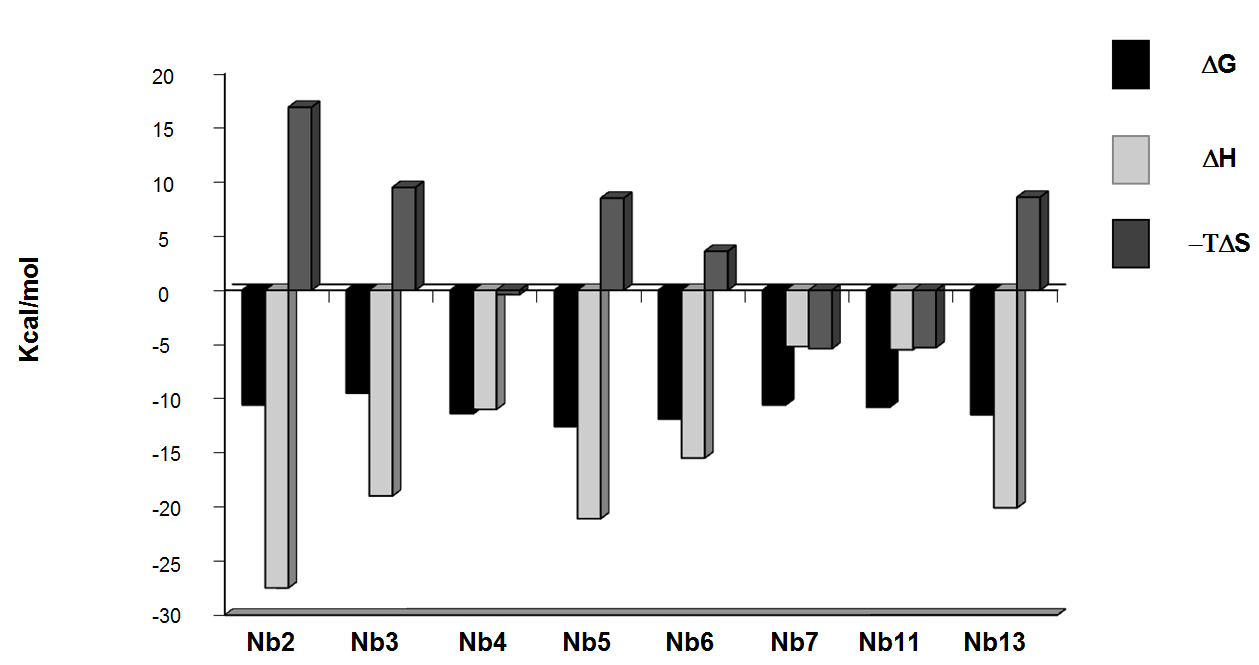
**

**Figure S2. Gibbs free energy, enthalpy and entropy changes associated with CapG-CAPNb interaction calculated from ITC measurements.** Most interactions are mainly enthalpy driven (light grey bar, negative ΔH) suggesting that binding involves a conformational change (induced fit). Reaction parameters for CAPNb4 imply a key and lock mechanism.

**
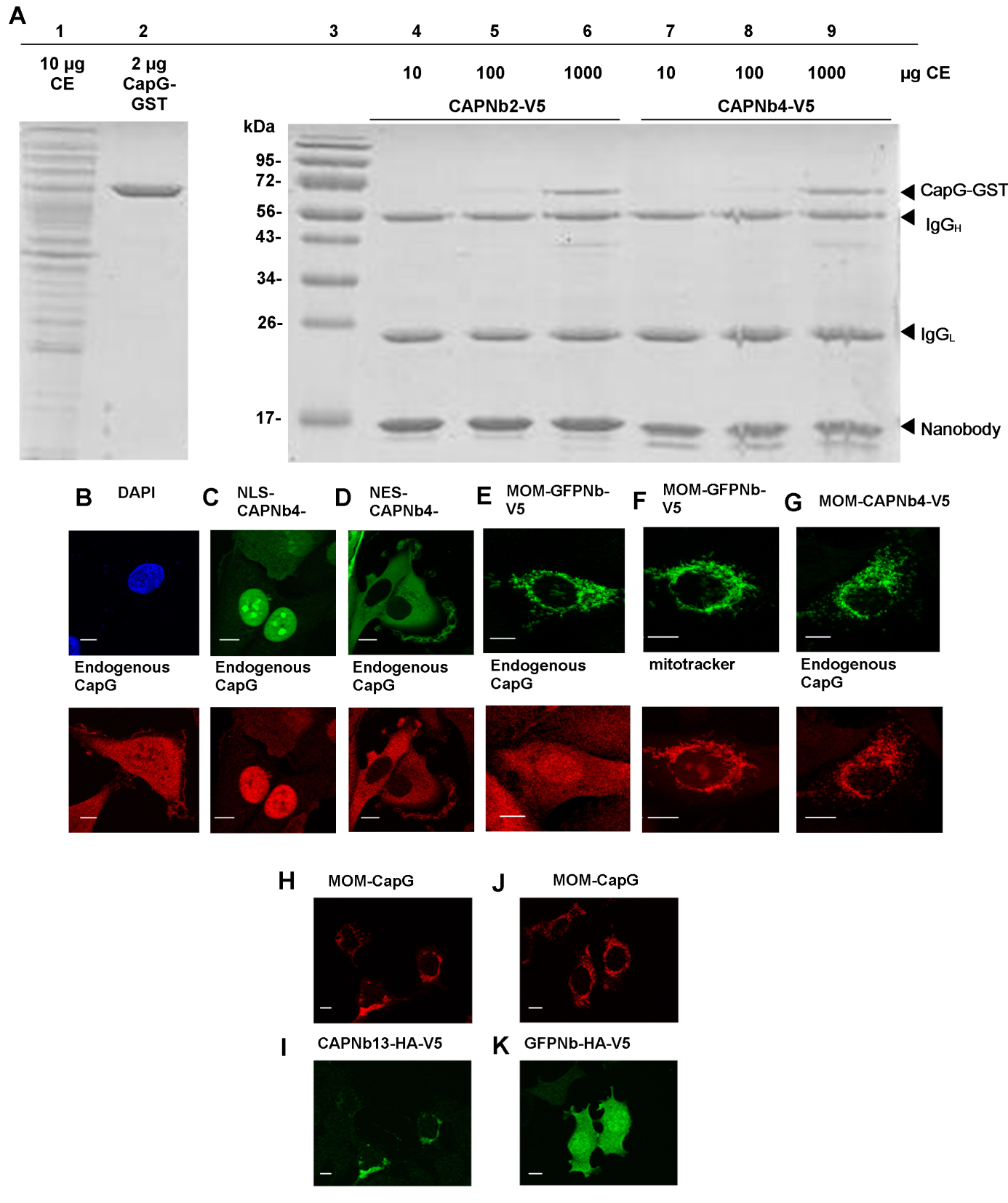
**

**Figure S3. Validation of CapG nanobodies in vitro and in vivo.**

(A) Pull-down of GST-CapG from *E. coli* extracts with selected nanobodies. Purified V5-tagged CAPNb2 or CAPNb4, immobilized on anti-V5 agarose, were incubated with the indicated amounts of *E.* *coli* extracts containing recombinant GST-CapG. Retained proteins were separated by SDS-PAGE and visualized by Coomassie staining. CE = crude extract. IgG_H_=V5 antibody heavy chain; IgG_L_=V5 antibody light chain. Size markers are indicated. (B-K) Endogenous CapG subcellular delocalization in MDA-MB-231 cells.

(B) DAPI stain (upper panel) and nucleo-cytoplasmic localization of endogenous CapG (lower panel).

(C) Nuclear localization of NLS-tagged CAPNb4 (upper panel) redirects CapG (lower panel) to the nucleus.

(D) CAPNb4 directs CapG to the cytoplasm when the nanobody is tagged with a nuclear export sequence (NES) from MAPKKK.

(E) The mitochondrial outer membrane (MOM) targeting sequence of TOM70, coupled to the GFP control nanobody, does not alter the localization of CapG.

(F) Mitotracker staining of MDA-MB-231 cells, a mitochondrion-selective probe, reveals co-localization with the MOM-tagged GFP nanobody.

(G) MOM-tagged CAPNb4 redistributes endogenous CapG to mitochondria.

(H) Mitochondrial localization of overexpressed CapG carrying a mitochondrial outer membrane (MOM) targeting sequence in transfected HEK293T cells.

(I) Enrichment of HA-tagged CAPNb13 at the outer mitochondrial membrane in HEK 293T cells expressing MOM-CapG.

(J-K) A GFP nanobody uniformly stains the cytoplasm/nucleus and is not targeted to mitochondria in HEK293T cells expressing MOM-CapG. Bar = 10 µm.

**
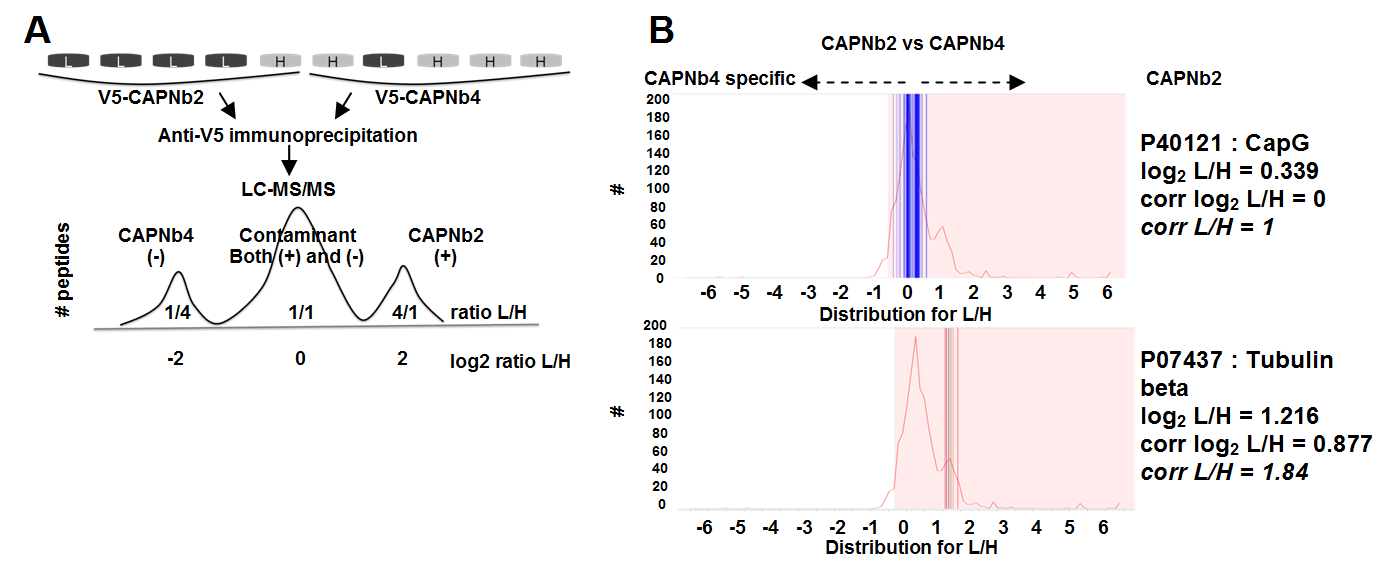
**

**C**

**
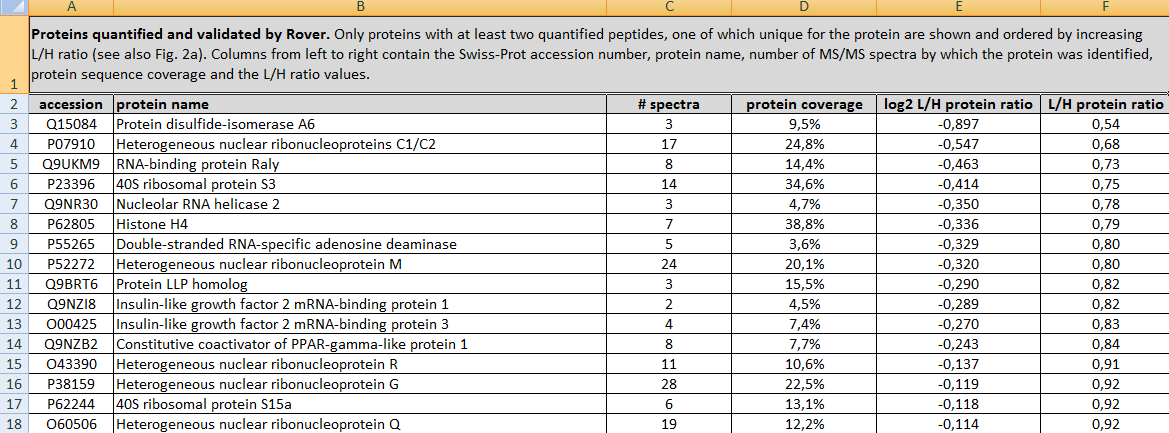
**

**Figure S4.** **Quantitative proteomics profiling**.

(A) Quantitative proteomics setup. CAPNb2 as well as CAPNb4 interactomes were obtained by immunoprecipitation from cell lysates using different mixtures of 'heavy' (H) and 'light' (L) labeled proteomes (4L:1H CAPNb2; 1L:4H CAPNb4) Immunoprecipitations were performed on proteomes of MDA-MB-231 cells and subjected to LC-MS/MS. (B) Tubulin peptides were enriched in the CAPNb2 interactome. Rover software screenshots are shown with vertical lines indicating L/H ratios of individual peptides from CapG (top) and tubulin beta (bottom). (C) Provided as an Excell sheet. First sheet: proteins quantified and validated by Rover. Only proteins with at least two quantified peptides, one of which unique for the protein are shown and ordered by increasing L/H ratio (see also Figure 3A). Columns from left to right contain the Swiss-Prot accession number, protein name, number of MS/MS spectra by which the protein was identified, protein sequence coverage and the L/H ratio values. Second sheet: list of all identified peptides. Peptides are ordered alphabetically by protein name and increasing start position. Columns from left to right contain the Swiss-Prot accession number, sequence of the identified peptide (Ace indicates N-terminal modification by acetyl, * indicates the presence of methionine sulfoxide (M*), pyro-glutamate (Q*), ^13^C_6_ lysine or ^13^C_6_ arginine residues), start and end position of the peptide in the protein sequence, m/z, charge (z), ppm, Mascot score and threshold by which the peptide was identified (99% confidence level), protein name and possible protein isoforms in which the same peptide sequence can be found.

**
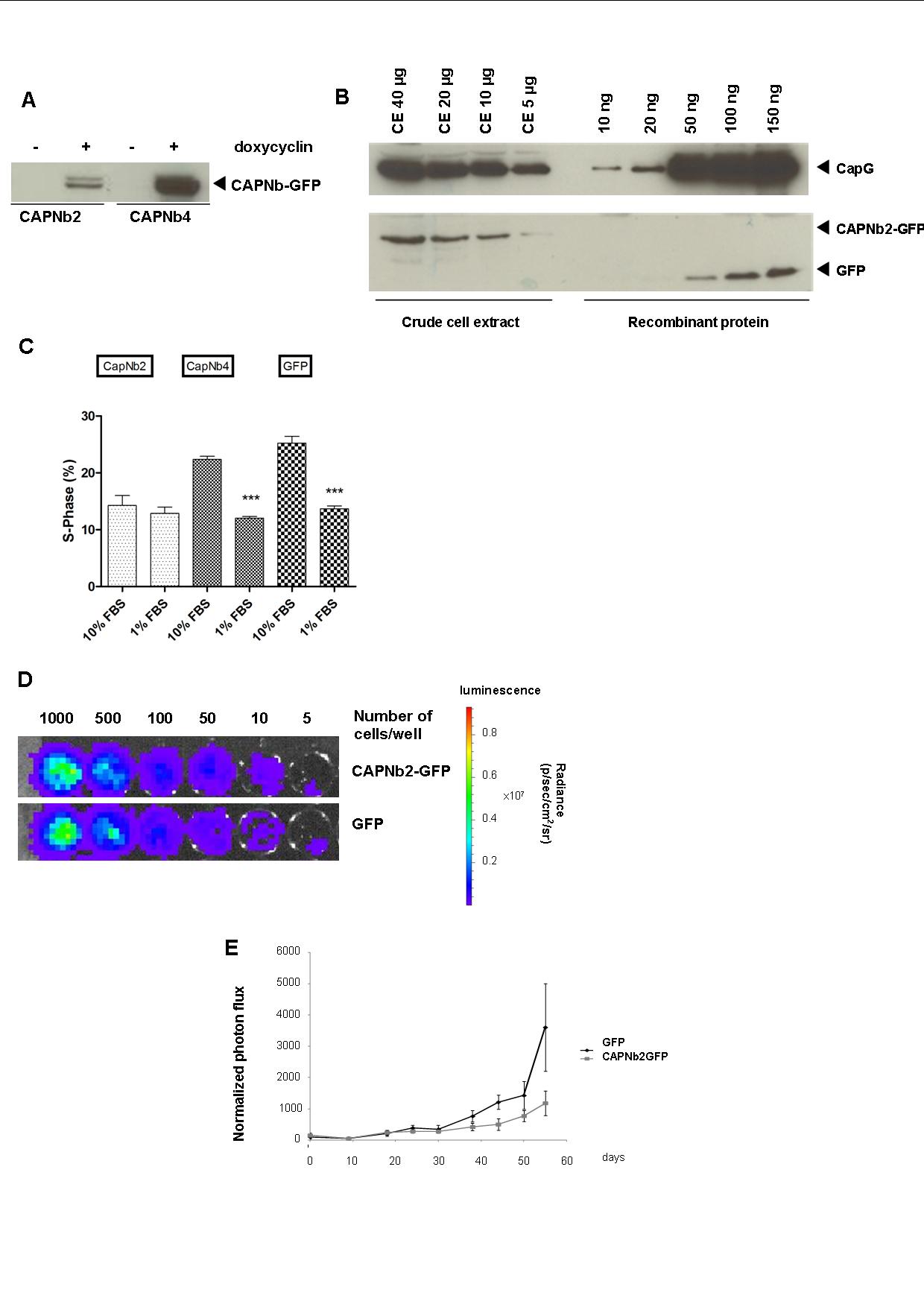
**

**Figure S5. Inducible CAPNb expression in breast cancer cells.**

(A) MDA-MB-231 cells were transduced with lentivirus encoding EGFP-CAPNb2 or EGFP-CAPNb4 and selected for with puromycin/G418. Cells were cultured in the absence or presence of 0.5 µg/ml doxycyclin, the highest concentration used in experiments. Following lysis and SDS-PAGE fractionation of 50 µg crude cell lysate, the blot was probed with anti-GFP antibody.

(B) Expression of CAPNb2 relative to endogenous CapG in MDA-MB-231 cells. Cell extracts were blotted and probed for endogenous CapG (top panel left, 5-40 µg CE = crude extract) or CAPNb2 (bottom panel left, 5-40 µg crude extract). Internal standards are shown at right; 10-150 ng recombinant human CapG, 10-150 ng recombinant GFP. Band intensity was used to calculate a CAPNb2 concentration that is equal to the endogenous CapG concentration.

(C) Changing the growth conditions from 10% FBS to 1% FBS and 1nM EGF (as used in migration and in vitro invasion experiments) causes a slight reduction in the S-phase population of MDA-MB-231 cells stably expressing CAPNb2 (10%) in comparison to CAPNb4 (47%) and the eGFP control (38%). However, under these conditions all three cell lines have comparable S-phase populations indicating a similar proliferation rate. ****P* < 0.001.

(D) Luciferase expression in MDA-MB-231 cells. GFP- or CAPNb2-GFP-expressing MDA-MB-231 breast cancer cells were transduced with a luciferase reporter. Serial dilutions of these cells in a 96 well plate showed comparable bioluminescence intensities.

(E) CAPNb2 reduces tumor volume. Primary tumor volume increase in mice orthotopically injected with control or CAPNb2-expressing breast cancer cells was measured by bioluminescence (normalized photon flux) over a period of 8 weeks. Error bars represent mean ± sem. *n*= 6~7.

**
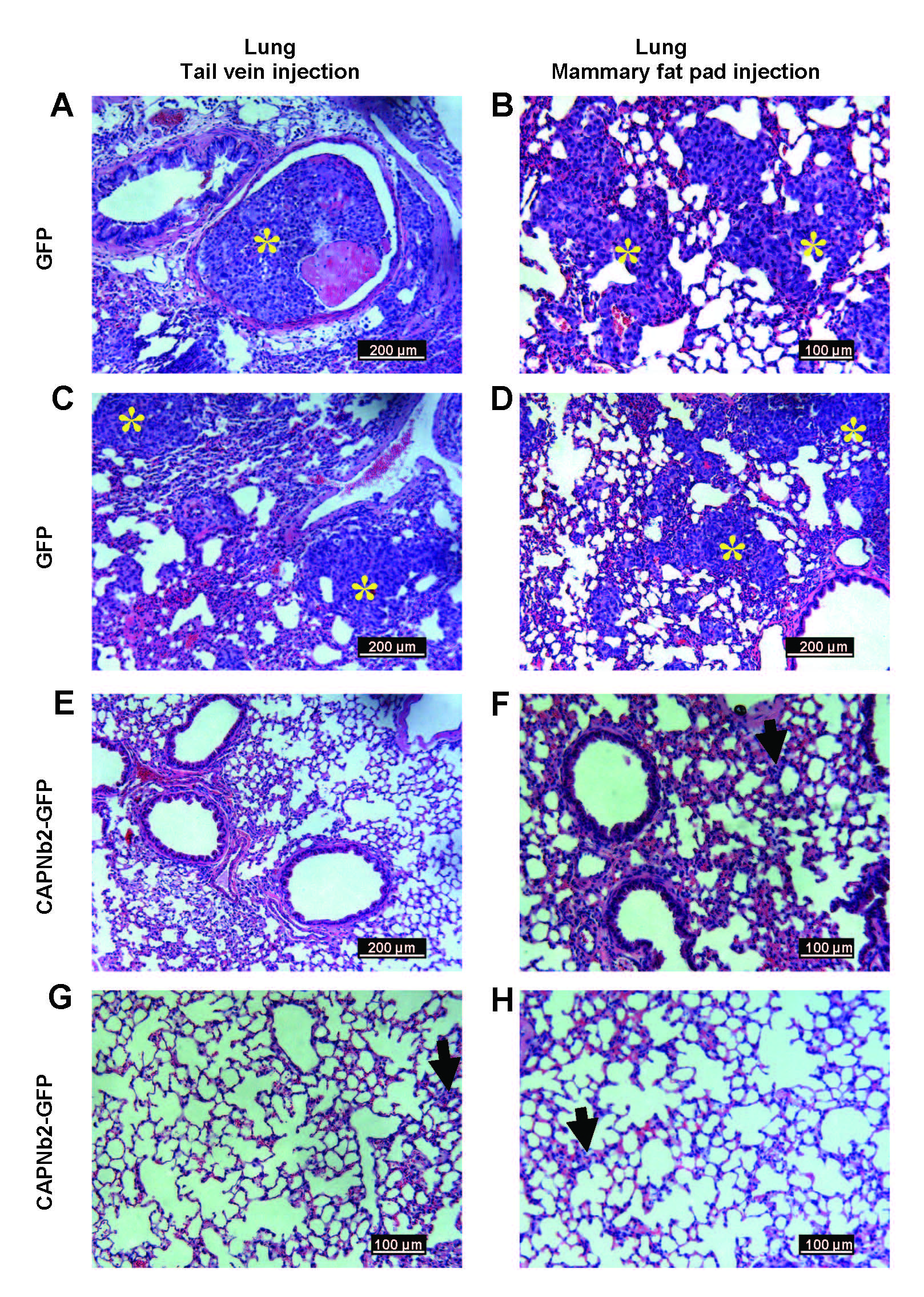
**

**Figure S6. Histological sections of** **lung metastatic lesions.** (A-D) Representative images showing hematoxylin and eosin staining of lung sections obtained from animals injected with MDA-MB-231-GFP cells in the tail vein (A,C) or in the mammary fat pads (B,D). Lesions are indicated by asterisks and are observed in a vessel (A) or in alveolar spaces (B-D). (E-H) Similar as in (A-D) but with lung sections obtained from mice injected with MDA-MB-231-CAPNb2 cells. Only small micrometastases are observed here (indicated by arrows).


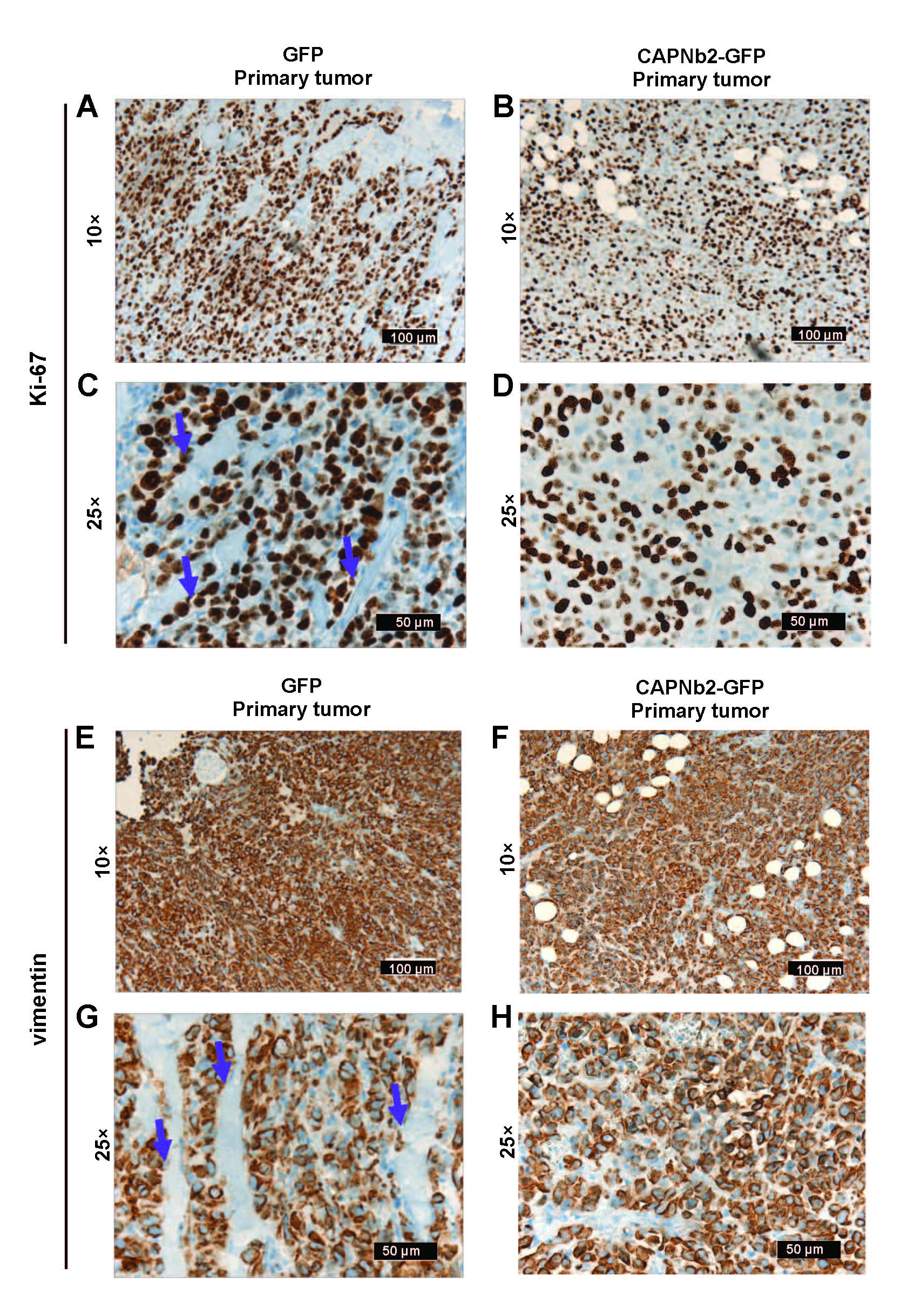


**Figure S7. Ki-67 and vimentin staining of primary tumor sections.**

(A-D) Ki-67 staining. Representative images showing proliferating cells in primary tumor sections obtained from animals injected with MDA-MB-231-GFP cells (A,C) or MDA-MB-231-CAPNb2 cells (B,D). Note the infiltration of breast cancer cells in muscle fibers in (C) (arrows). (E-H) Vimentin staining**.** Representative images showing vimentin-positive MDA cells in primary tumor sections obtained from animals injected with MDA-MB-231-GFP cells (E,G) or MDA-MB-231-CAPNb2 cells (F,H). Infiltration of breast cancer cells in muscle fibers in (G) is marked by arrows.

**References**

1. Impens F, Colaert N, Helsens K, Ghesquiere B, Timmerman E, De Bock PJ, Chain BM, Vandekerckhove J, Gevaert K: **A quantitative proteomics design for systematic identification of protease cleavage events**. *Mol Cell Proteomics* 2010, **9**(10):2327-2333.

2. Kall L, Storey JD, MacCoss MJ, Noble WS: **Assigning significance to peptides identified by tandem mass spectrometry using decoy databases**. *J Proteome Res* 2008, **7**(1):29-34.

3. Colaert N, Helsens K, Impens F, Vandekerckhove J, Gevaert K: **Rover: a tool to visualize and validate quantitative proteomics data from different sources**. *Proteomics* 2010, **10**(6):1226-1229.

4. Helsens K, Colaert N, Barsnes H, Muth T, Flikka K, Staes A, Timmerman E, Wortelkamp S, Sickmann A, Vandekerckhove J *et al*: **ms_lims, a simple yet powerful open source laboratory information management system for MS-driven proteomics**. *Proteomics* 2010, **10**(6):1261-1264.

5. De Corte V, Van Impe K, Bruyneel E, Boucherie C, Mareel M, Vandekerckhove J, Gettemans J: **Increased importin-beta-dependent nuclear import of the actin modulating protein CapG promotes cell invasion**. *J Cell Sci* 2004, **117**(Pt 22):5283-5292.

6. Hubert T, Van Impe K, Vandekerckhove J, Gettemans J: **The actin-capping protein CapG localizes to microtubule-dependent organelles during the cell cycle**. *Biochem Biophys Res Commun* 2009, **380**(1):166-170.

7. Van Impe K, Hubert T, De Corte V, Vanloo B, Boucherie C, Vandekerckhove J, Gettemans J: **A new role for nuclear transport factor 2 and Ran: nuclear import of CapG**. *Traffic* 2008, **9**(5):695-707.
